# Laser ablation of tree-ring isotopes: pinpoint precision

**DOI:** 10.1093/treephys/tpad017

**Published:** 2023-02-20

**Authors:** John Marshall

**Affiliations:** Forest Ecology and Management, Swedish University of Agricultural Sciences, Skogmarksgränd 17, 90183 Umeå, Sweden; Leibniz-Centre for Agricultural Landscape Research (ZALF) e.V., Eberswalder Str. 84, 15374 Müncheberg, Germany; Global Change Research Institute CAS, Bělidla 986/4a, 60300 Brno, Czechia

This scientific commentary refers to ‘Progress in high-resolution isotope-ratio analysis of tree rings’ by Saurer et al. (https://doi.org/10.1093/treephys/tpac141).

Trees’ annual rings provide a remarkable record of the past, dated precisely by the annual cycle of xylem cell formation. Although dendrochronology often focuses on ring widths, there have been many studies exploring the chemistry of the material comprising the rings as well. Particularly interesting in this respect is the isotopic composition of the cell-wall material, which provides detailed information about the weather conditions and the physiological state of the tree during the period of growth (McCarroll and Loader 2004, Siegwolf et al. 2022). These quality data do not come easily or cheaply, however. Isotope analysis can be tricky, and the manual splitting of rings is tedious. Therefore, any improvement in precision or ease of sampling would be most welcome.

Nearly 20 years ago, laser ablation seemed to offer such an opportunity (Schulze et al. 2004), however its initial promise has been slow to mature. Its challenges have been addressed in the recent paper of Saurer et al. (2022), which describes two systems in regular use at WSL in Switzerland and LUKE in Finland. The paper includes detailed assessments of system performance and a comparison of alternatives for sample preparation. Considered alongside other recent advances (e.g., Loader et al. 2017), the time may finally be ripe for efficient adoption and standardization of this technique.

‘Ablation’ refers to the removal of material from the surface, often implying gradual loss of particles or fragments, but the products may also include escaping gases and liquids. In the systems described here, ablation breaks molecules free from a point on the surface of a tree-ring sample and delivers the ablated material to a ratioing mass spectrometer for isotope analysis. It is a simple way to feed the instrument with material from a known location on the wood sample. The ablated material is gathered into a helium stream and transferred to an oven with an abundance of oxygen, where carbon compounds are converted quantitatively to CO_2_. The CO_2_ is then collected, concentrated and cleaned. The clean CO_2_ pulse is then transferred to a ratioing mass spectrometer, where natural abundance of the stable isotopes can be precisely determined.

One advantage of this technique is that the tight focus of a laser provides precise control of the sampled position on the wood surface. This would be advantageous in any discipline, but it is especially so in dendrochronology, where space implies time. Because a tree ring is produced from the inside outward over a growing season (Cuny et al. 2013), position in the ring can be used to estimate the date of deposition of the material being analysed. This capacity has distinct advantages when seeking short-term events, e.g., a short drought period in the middle of a growing season (Schiestl-Aalto et al. 2021). It is also possible to detect these events using a microtome to slice an annual ring into thin sections (Loader et al. 1995, Helle and Schleser 2004), but the microtome technique is difficult to apply. More common is to split out whole rings from a core without attempting to subsample them, or perhaps to divide the rings into earlywood and latewood sections (e.g., Marshall and Monserud 1996). But even this becomes difficult when rings are narrow, as frequently observed in harsh climates and on large-diameter trees. A second advantage of the laser is that it is relatively noninvasive (Loader et al. 2017), charring points on the surface of a wood core without influencing the remainder of the sample. For this reason, it can be applied to high-value, or even irreplaceable, wood samples that could otherwise not be measured.

Saurer et al. also compared sample preparation methods. For decades, tree-ring isotope analysis has favoured cellulose extracted from the rings, especially for climate reconstruction. Cellulose is preferred because it is a simple chain of glucose molecules, free of isotopic interferences due to the presence of other compounds. Especially problematic are resins and lipids, but lignin also displays consistent isotopic deviations from cellulose (Harlow et al. 2006). On the other hand, pure cellulose lacks the rigidity of whole wood, which makes it difficult to mount for the laser (Schulze et al. 2004). Nevertheless, these authors managed to extract cellulose from intact wedges of wood and compared the isotopic composition with cores extracted to remove lipids and resins, and with whole wood. The results provide a laser-specific translation between traditional cellulose to the lipid-extracted wood more suitable for the laser.

Critical to the operation of these devices are the physicochemical events at the surface of the laser-charred crater. This is important because the samples are small and the precision of the isotope measurement is influenced by sample size. Saurer and colleagues performed several experiments with various burn parameters to generate different sample sizes, presenting minima for their systems and exemplifying how such tests could be performed on other systems. These results are presented in terms of beam strength, which describes the amount of material being ablated and delivered to the instrument. Earlier studies have assumed that ablation produces particles (e.g., Schulze et al. 2004), which would be carried into the gas stream and combusted in the furnace downstream. However, the new data suggest that the product of the laser strike is almost exclusively CO, carbon monoxide gas, which is later converted to CO_2_ in the oven.

The production of CO is important because it presents opportunities for isotopic analysis of the oxygen as well as the carbon in the sample (Wieser and Brand 1999). There is no oxygen gas in the laser chamber so the only source of oxygen for the CO is the wood itself. Traditional oxygen isotope analysis of ground samples relies on the production of CO at high temperatures under anoxic conditions by pyrolysis (Saurer et al. 1998). These new results suggest that the wood may similarly be pyrolyzed to CO at the site of the laser strike. Oxygen isotopes are frequently used in climate reconstructions (Büntgen et al. 2021) as well as physiological reconstructions, where it can be used to infer rooting depths and stomatal behaviour (Marshall and Monserud 2006, Barbour 2007). Although laser-ablation was long ago applied to δ^18^O of tooth enamel (Cerling and Sharp 1996), the author is not aware of a large-scale application to tree rings. These results make such tests a priority.

The manuscript also noted that the beam strength, which measures the amount of material ablated, was correlated with known patterns of wood density. Denser material would release more CO_2_ if the laser ablated a similar wood volume. This is useful because, in some trees, drought events lead to short-term increases in wood density within the rings (Rigling et al. 2001, Battipaglia et al. 2014). Such density fluctuations are typically measured with X-ray absorption, but these results suggest that laser ablation might be used as well, perhaps in tandem with stable-isotope analysis.

The manuscript closes with an example of ^13^C-labelling experiments conducted in a pine forest. The distribution of the label in the tree rings shows a distinct anomaly in portions of the wood produced after the labelling was conducted. In some trees, the labelling was blurred, perhaps by resin deposition or reserve accumulation and later utilization. The blurring was possible to detect only because the precision of the laser shots was so high. The laser ablation system provides great opportunities for pulse–chase studies with stable-isotope labels (Högberg et al. 2008, Gao et al. 2021), which will lead to new insights into the controls, timing and precise placement of carbon into wood across growing seasons and under changing conditions. Perhaps the same will be possible with oxygen isotopes in the near future.
